# Orientations and Proximities of the Extracellular Ends of Transmembrane Helices S0 and S4 in Open and Closed BK Potassium Channels

**DOI:** 10.1371/journal.pone.0058335

**Published:** 2013-03-05

**Authors:** Xiaowei Niu, Guoxia Liu, Roland S. Wu, Neelesh Chudasama, Sergey I. Zakharov, Arthur Karlin, Steven O. Marx

**Affiliations:** 1 From the Center for Molecular Recognition, Departments of Biochemistry, Physiology, and Neurology, College of Physicians and Surgeons, Columbia University, New York, New York, United States of America; 2 Division of Cardiology, Department of Medicine, College of Physicians and Surgeons, Columbia University, New York, New York, United States of America; 3 Department of Pharmacology, College of Physicians and Surgeons, Columbia University, New York, New York, United States of America; Virginia Commonwealth University, United States of America

## Abstract

The large-conductance potassium channel (BK) α subunit contains a transmembrane (TM) helix S0 preceding the canonical TM helices S1 through S6. S0 lies between S4 and the TM2 helix of the regulatory β1 subunit. Pairs of Cys were substituted in the first helical turns in the membrane of BK α S0 and S4 and in β1 TM2. One such pair, W22C in S0 and W203C in S4, was 95% crosslinked endogenously. Under voltage-clamp conditions in outside-out patches, this crosslink was reduced by DTT and reoxidized by a membrane-impermeant bis-quaternary ammonium derivative of diamide. The rate constants for this reoxidation were not significantly different in the open and closed states of the channel. Thus, these two residues are approximately equally close in the two states. In addition, 90% crosslinking of a second pair, R20C in S0 and W203C in S4, had no effect on the V_50_ for opening. Taken together, these findings indicate that separation between residues at the extracellular ends of S0 and S4 is not required for voltage-sensor activation. On the contrary, even though W22C and W203C were equally likely to form a disulfide in the activated and deactivated states, relative immobilization by crosslinking of these two residues favored the activated state. Furthermore, the efficiency of recrosslinking of W22C and W203C on the cell surface was greater in the presence of the β1 subunit than in its absence, consistent with β1 acting through S0 to stabilize its immobilization relative to α S4.

## Introduction

The large-conductance potassium (BK) channel is a tetramer of α (Slo1) subunits and up to four auxiliary β subunits. Membrane depolarization and increased intracellular Ca^2+^ cooperatively activate the channel [1]–[3]. K^+^ current through the open BK channel shifts the membrane potential negatively. In smooth muscle and nerve cells this hyperpolarizing shift suppresses voltage-dependent Ca^2+^ channel activity, affecting negative feedback regulation of intracellular [Ca^2+^]. The α subunit contains a voltage-sensor domain (VSD) formed by a unique N-terminal, transmembrane (TM) helix S0 [4] followed by four TM helices S1- S4, versions of which are found in all voltage-dependent cation channels [5], [6] and a pore domain. As in all other K^+^ channels, this is formed by the TM helices S5 and S6 separated by a reentrant pore helix and selectivity-filter containing loop. The remaining two-thirds of the α subunit are cytoplasmic and contain two Ca^2+^-binding RCK domains [7]–[9]. In the tetrameric complex, the cytoplasmic domains form a gating ring that transduces Ca^2+^ binding into a stabilization of the open state of the pore [10]–[12].

The responses of BK channels to voltage and Ca^2+^ are tuned by their associations with tissue-specific, auxiliary β subunits of which there are four major types, β1 through β4 [13]–[17]. The β subunits have short cytoplasmic N-terminal and C-terminal tails and two TM helices TM1 and TM2 connected by an approximately 100-residue-long, extracellular loop. In smooth muscle, BK α associates with the β1 subunit, which at [Ca^2+^] >1 μM shifts the V_50_ for channel activation negatively towards the resting potential, priming it for activation by increases in intracellular Ca^2+^ [18]–[21]. In addition, the association of β1 with αslows both activation and deactivation of the channel.

Previously, we showed that the extracellular ends of S0 and S4 are contiguous and that TM1 and TM2 of both β1 and β4 dock between adjacent αVSDs. At least at their extracellular ends, TM2 is next to S0 of one VSD, and TM1 is next to S1 and S2 of the adjacent VSD [22]–[25]. Our initial approach was to determine the extent of endogenous disulfide bond formation between Cys substituted for the first four residues predicted to just flank the extracellular ends of the TM helices. A surprising result was that nearly complete disulfide crosslinking between particular cysteines in the flanks of S0 and S4 (e.g., R17C and R201C) had remarkably small effects on V_50_, k_act_ , and k_deact_ [22]. Although not all crosslinks between the flanks of S0 and S4 had small effects, that some did seemed inconsistent with more than a modest relative displacement during activation of the extracellular ends of S0 and S4.

Because of the possibility that there might be sufficient flexibility in the flanks to confound both our structural and functional inferences, we mutated to Cys in pairs the four residues in the first helical turns in the membrane of S0, S4 and TM2. Compared to Cys in the flanks, these Cys in the membrane would likely be in a more constrained helical structure, albeit less accessible to water and to reagents and hence less reactive. Because of the structural constraints, disulfide crosslinking between these helices should strongly perturb activation if it involves relative movements of their extracellular ends. Recently, such relative movement was inferred from voltage-dependent perturbation of the fluorescence of fluorophore-labeled BK α [26].

We now describe the functional consequences of crosslinks of substituted Cys in the first helical turns in the membrane of S0 and S4 and the effects of the functional state of the VSD on the rates of crosslinking. We also demonstrate that the efficiency of recrosslinking between cysteines in S0 and S4 on the cell surface is greater in the presence of the β1 subunit than in its absence, consistent with β1 acting through S0 to stabilize its interaction with S4.

## Materials and Methods

### Constructs

**Figure 1:**
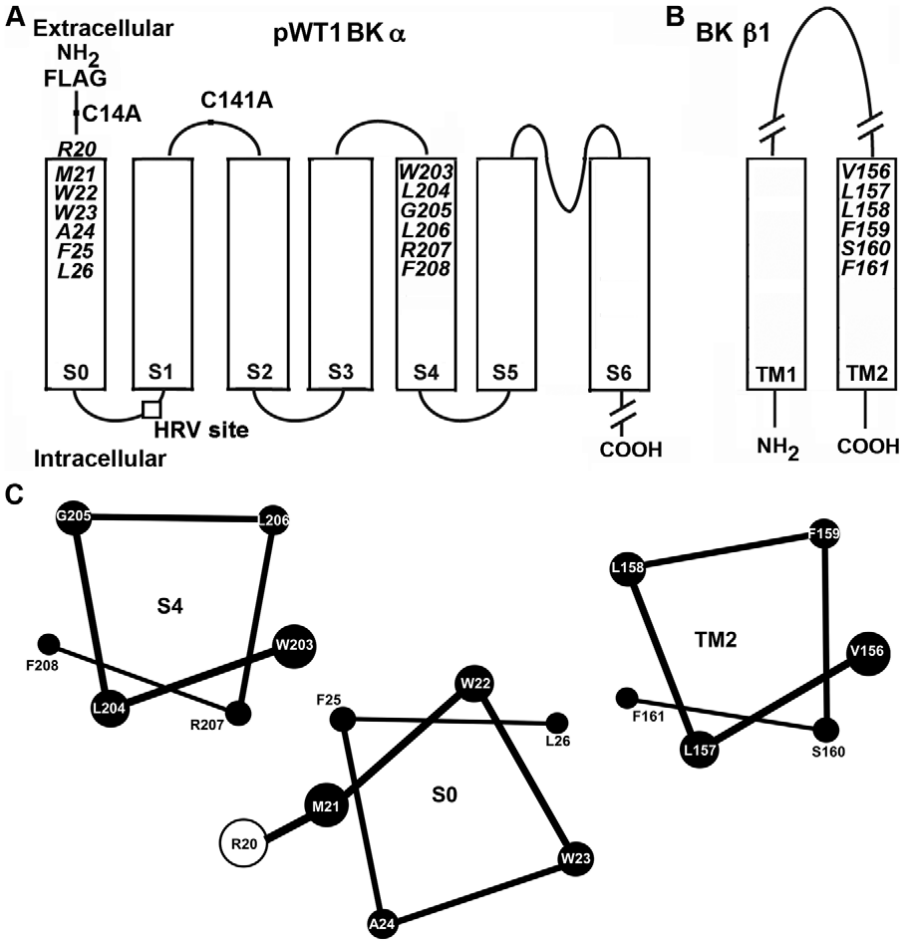
Membrane topology of BK α and β1 subunits. (A) Mouse BK α residues mutated to Cys in the first two turns of S0 and S4. An HRV-3C protease cleavage site was inserted in the S0-S1 loop (box), the two native extracellular Cys, C14 and C141, were mutated to Ala, and a FLAG-epitope (MDYKDDDDKSPGDS) was added to its N-terminus. This construct is termed pWT1 α. (B) Mouse BK β1 residues mutated to Cys in the first two turns of TM2. (C) The residues at the extracellular ends of S0, S4, and TM2 in the membrane are represented as ideal αhelices, as viewed from the extracellular side. The relative positions and orientations of the helices optimize the average observed endogenous crosslinking between Cys.

Mutants of mouse BK αsubunit (mSlo1, KCNMA1, Genbank/EMBL/DDBJ accession no. NM_010610) were generated in a pseudo-wild-type α pWT1 αcontaining the two extracellular Cys, C14 and C141, mutated to Ala, an N-terminal FLAG epitope (MDYKDDDDKSPGDS), and the human rhinovirus (HRV)-3C protease-consensus-cleavage site, LEVLFQGP, inserted in the S0-S1 loop. This was created by the mutation A89L and the insertion of LFQGP between Val91 and Gly92 [22], [25] (Fig. 1A). Cys-substitutions in mouse β1 subunit (KCNMB1) were made in a pWT β1, which contained mutations C18A and C26A.

### Expression of α and β1 constructs

HEK293 cells were transfected with the appropriate constructs of pWT1 α alone or of pWT1 α and pWT β1. To determine the extent of crosslinking, we surface biotinylated the intact cells for 10 min with 1 mM sulfosuccinimidyl-6-(biotinamido) hexanoate (sulfoNHS-LC-biotin; ThermoFisher Scientific) in DPBS, pH 7.4, quenched the reaction with glycine methyl ester, and solubilized the cells in lysis buffer (1% Triton X-100, 150 mM NaCl, 50 mM Tris, 1 mM EDTA, and protease inhibitors) containing 2 mM *N*-ethylmaleimide. The lysate was mixed with Ultralink Immobilized NeutrAvidin Plus beads (Thermo-Fisher Scientific), which were washed extensively, and the bound biotinylated proteins were eluted in 4 M urea in 2% SDS at 100°C [22], [23].

### Intrasubunit crosslinking of α

The extent of crosslinking between Cys-substituted in S0 and S4 in the same subunit of pWT1 α was determined as previously described [22]. In brief, biotinylated-proteins were captured on NeutraAvidin Ultralink beads. The beads were stirred with HRV-3C protease (EMD) overnight at 4°C. Proteins were eluted in 4 M urea in 2% SDS at 100°C. One-half of each sample was reduced with 10 mM DTT (pH 8.0), 20 min at 50°C. Aliquots of unreduced and reduced samples were electrophoresed, transferred to nitrocellulose, and immunoblotted with anti-BK α-C-terminal-epitope antibody (BD Biosciences) and horseradish-peroxidase (HRP)-conjugated secondary antibody. Chemiluminescence was recorded with a CCD camera (Carestream) and quantitated with ImageQuant software (Molecular Dynamics). The fraction of crosslinked α in the unreduced aliquot was corrected for the efficiency of protease cleavage, determined from the DTT-reduced aliquot [22], [25].

### Crosslinking of α and β subunits

We determined the extent of crosslinking between Cys-substituted pWT1 α and pWT β1 as previously described [23]–[25]. We calculated the extent of crosslinking from the integrated luminescence from the α-β band at apparent mass ∼160 kDa, divided by the sum of the integrated luminescence of the bands at ∼130 kDa (α) and ∼160 kDa (α β).

### Reduction of disulfides and reoxidation of thiols

Transfected HEK293 cells were surface-biotinylated as above. Disulfides were reduced with 10 mM dithiothreitol (DTT) in a solution containing 137 mM NaCl, 2.7 mM KCl, 0.1 mM CaCl_2_, 0.1 mM MgCl_2_, and 40 mM HEPES (pH 8.0). The cells were washed with DPBS. Cys thiols were oxidized with 40 μM 4,4’-(azodicarbonyl)-bis-[1,1-dimethylpiperazinium, diiodide] [QPD], a bis-quaternary ammonium, piperazinium diamide [22], [27] in 137 mM NaCl, 2.7 mM KCl, 0.9 mM CaCl_2_, and 0.49 mM MgCl_2_, 10 mM MOPS (pH 7.2). Cells were washed and lysed as detailed above.

### Electrophysiology

Macroscopic currents were recorded from HEK293 cells in the outside-out-patch-clamp configuration, as described previously [25]. The Cys-substitutions were created in the pWT1 α background. The V_50_ for pWT1 α was shifted in the depolarizing direction by ∼40 mV compared to WT α. For the measurement of conductance as a function of membrane potential (G-V data), macroscopic currents were activated by depolarizing steps from a holding potential of −100 mV and deactivated by repolarization to −100 mV, at which deactivating tail currents were measured. G-V data were fitted with a Boltzmann function. Time constants for activation (step to +80 mV) and deactivation (return to −100 mV) were estimated from exponential fits of the macroscopic currents with Clampfit (MDS Analytical Technologies). The bath solution was 150 mM KCl, 5 mM TES, and 1 mM MgCl_2_ (pH 7.5). The pipette solution contained 0–100 μM free Ca^2+^ in 150 mM KCl, 1 mM HEDTA, 5 mM TES (pH 7.0). The free Ca^2+^ concentration was calculated using the Max Chelator program.

The functional effects of the reduction and of the re-oxidation of the disulfide bond were determined after perfusion of the patch with 10 mM DTT (5 min) in 150 mM KCl, 5 mM TES, 5 mM EGTA (pH 7.5) or with 40 μM QPD (2 min) in the same buffer, respectively, through a fast perfusion system (SF-77B, Warner Instrument). The patches were held at −100 mV for reoxidation in the closed state and at +80 mV for reoxidation in the open state. The EGTA in the perfusion solution chelated any contaminating divalent metal ions.

The kinetics of reformation of disulfide bond between W22C and W203C was determined during the application of 40 μM QPD, while holding the membrane potential for 1890 ms at either −100 mV or +80 mV. After 50 ms at −120 mV, the patch was depolarized to +20 mV for 30 ms and hyperpolarized to −120 mV for 30 ms, during which the tail current was recorded. This cycle was repeated every 2 s. The data were fit with a single exponential function, and the means of the rate constants from the least-squares fits of 5 independent experiments were determined. The pipette solution contained 10 μM Ca^2+^.

### Statistical analysis

A one-way ANOVA was used for multiple comparisons followed by Tukey post-hoc test if the null hypothesis was rejected. An unpaired Student’s t test was utilized for comparison of two separate groups. Differences were considered statistically significant at P<0.05. All statistical analysis was performed using Graphpad Prism 6.

## Results

### Functional effects of crosslinks between S0 and S4

BK α S0 and S4, as well as BK β1 TM2, are predicted to be membrane-embedded α-helices. We mutated to Cys, one per helix, the six residues closest to their extracellular ends (Fig. 1A,B) and expressed these double-mutant α subunits in HEK293 cells. Similarly, we co-expressed single-Cys mutants of α with single-Cys mutants of β1 TM2. We determined the extent to which these Cys formed crosslinks endogenously; i.e., without the addition of any reagents. We have argued previously [22] that this crosslinking occurs mainly during subunit folding and assembly in the endoplasmic reticulum [28]. The extents of crosslinking and the functional effects of the crosslinks were determined exclusively on BK channel complexes that were transported to the cell surface. Previously, we determined the extents of endogenous disulfide crosslinking in sixteen pairs of Cys in S0 and S4 and in sixteen pairs in S0 and TM2 [25]. We now describe the susceptibilities of these surface-expressed, disulfide-crosslinked channels to reduction by DTT and to reoxidation by an impermeant, bisquaternary-ammonium diamide (QPD). We also report the functional consequences of these crosslinks as well as of the mutations to Cys per se.

**Figure 2:**
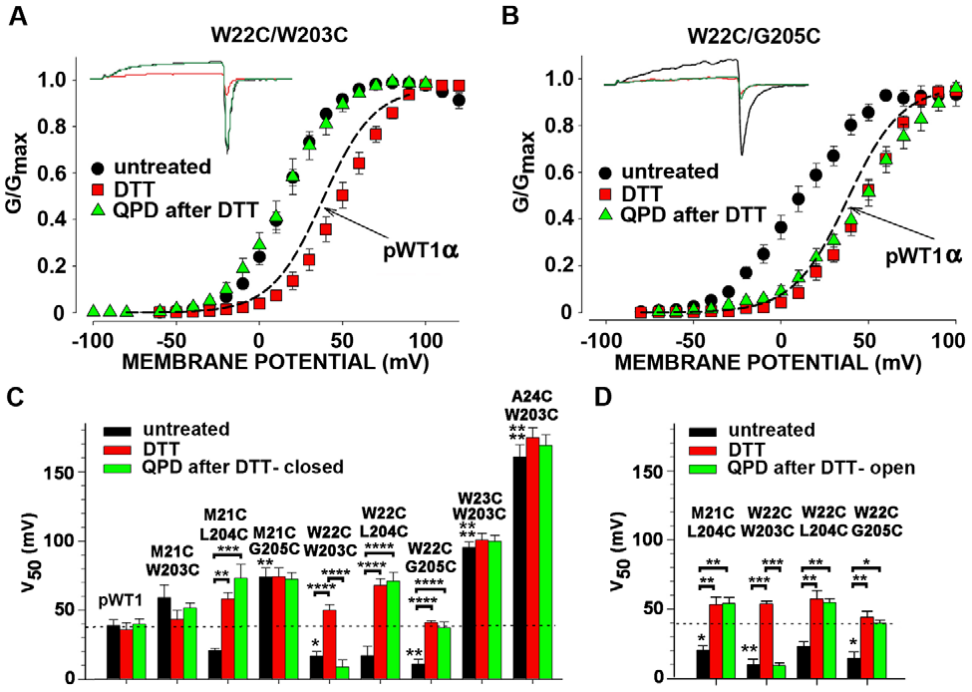
Effects on V_50_ of endogenously formed S0-S4 disulfide bonds, their reduction, and their QPD-induced reformation at the cell surface. (A, B) Macroscopic currents (insets) and normalized G-V curves of untreated cells expressing W22C/W203C (A) or W22C/G205C (B), after treatment with 10 mM DTT (pH 7.5) for 5 min, and after subsequent treatment with 40 μM QPD (pH 7.5) for 2 min. Recordings were from outside-out macropatches with 10 μM Ca^2+^ inside the pipette. At each potential, the mean relative conductance averaged from several cells is plotted. The mean G-V curve for pWT1 α is shown as a dashed-line. (C) Mean V_50_ ± SD of the V_50_s from the individual fits of the Boltzmann equation to the currents from each cell. The V_50_s were determined after endogenous disulfide crosslinking (black bars), after subsequent DTT (red bars), and finally after 40 μM QPD (green bars). The mean V_50_ for pWT1 α is shown as a dashed-line. In C, the macropatches were held at −100 mV (closed state) during the QPD-induced reoxidation. N = 3–11. (D) As C, except that the patches were held at +80 mV (open state) during the application of QPD. The mean V_50_ for pWT1 α is shown as a dashed-line. * P<0.05, **P<0.01, *** P<0.001, **** P< 0.0001 by one-way Anova followed by Tukey’s post-hoc analysis for multiple comparisons between brackets. Without brackets, comparison to pWT1 αby one-way Anova followed by Tukey’s post-hoc analysis.

For the eight double-Cys α mutants that exhibited an extent of endogenous crosslinking of at least 45% [25], we determined the effects of the crosslinks on the dependence of channel conductance on the membrane potential (G-V curve). In four of the eight pairs, the G-V curves were shifted to the left compared to the G-V curve of pWT1 α. This is illustrated by the G-V curves of α W22C/W203C and of α W22C/G205C (Fig. 2A,B). For these two, the mean V_50_s were 22 mV and 28 mV were more negative than the V_50_ for pWT1 α (Fig. 2C; P<0.05 and P<0.01 respectively,). Similarly, the V_50_s of M21C/L204C and W22C/L204C were shifted negatively by about 20 mV. Moreover, for each of the four pairs, reduction by DTT shifted the G-V curves back to or even a little to the right of the G-V curve of pWT1 α. In these cases, the crosslink *per se* stabilized the open state compared to the closed state; i.e., less electrostatic energy is needed to open the channel of these crosslinked mutant αs compared to pWT1 α. In only one case, α W22C/W203C was the positive shift in V_50_ due to reduction reversed by the impermeant oxidizing agent QPD (Fig. 2C; P<0.0001, DTT vs. QPD). Both DTT and QPD were applied to outside-out patches at a holding potential of −80 mV, with the channels predominantly in a closed state.

For the other four disulfide-crosslinked α mutants, the G-V curves and the V_50_s were shifted in a positive direction (Fig. 2C). In none of these did DTT cause a statistically significant shift back towards the V_50_ of pWT1 α, even though we found that the disulfides were reduced (see below), implying that the mutations themselves were sufficient cause for the positive shift in V_50_.

**Figure 3:**
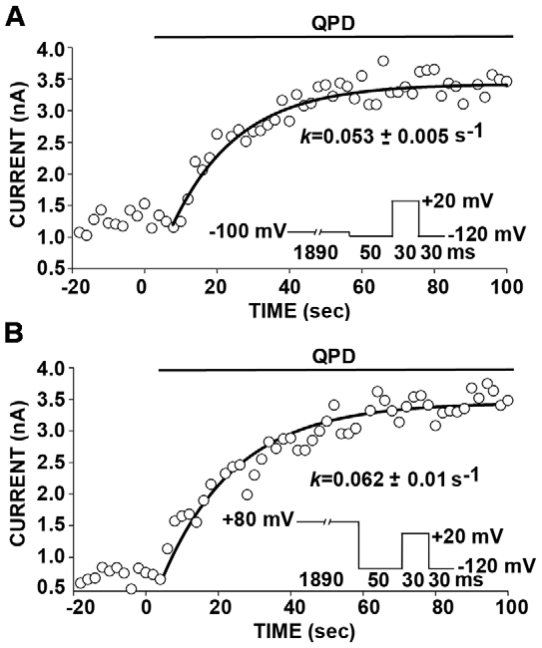
Kinetics of reformation of disulfide bond between W22C and W203C in the closed state (A) and in the open state (B). Outside-out patches were bathed in 10 mM DTT (pH 7.5) for 5 min. During the subsequent application of 40 μM QPD, membrane potential was held for 1890 ms at either −100 mV (A) or +80 mV (B). After 50 ms at −120 mV, the patch was depolarized to +20 mV for 30 ms and hyperpolarized to −120 mV for 30 ms, during which the tail current was recorded. This cycle, represented in the insets, was repeated every 2 s. The peak amplitudes of the tail currents are plotted against elapsed time. The data were fit with a single exponential function. The means of the rate constants from the least-squares fits of 5 independent experiments are given under the curves. The pipette solution contained 10 μM Ca^2+^. N =  4 for closed state and n = 5 for open state. P =  not significant by unpaired Student’s t-test.

In the one case, α W22C/W203C, in which QPD shifted the G-V curve back negatively, we determined whether the extents or the rate constants for the QPD reaction were different in the open and closed states of the channel. We found that the extents of disulfide formation were the same after 2 minutes of application of 40 μM QPD in the open and closed states (Fig. 2D). More telling was the kinetics of the QPD reactions in the two states. The effects of QPD applied at −100 mV (deactivated state) or at + 80 (activated state) on DTT-reduced W22C/W203C were recorded every 2 s (Fig. 3). The negative shift in the V_50_ as the disulfide reformed was reflected in the increased current at +20 mV. There was no statistical difference in the rate constants determined in the deactivated state (Fig. 3A) and in the activated state (Fig. 3B).

### Biochemical determination of the extents of reduction and reformation of disulfides between BK α S0 and S4

**Figure 4:**
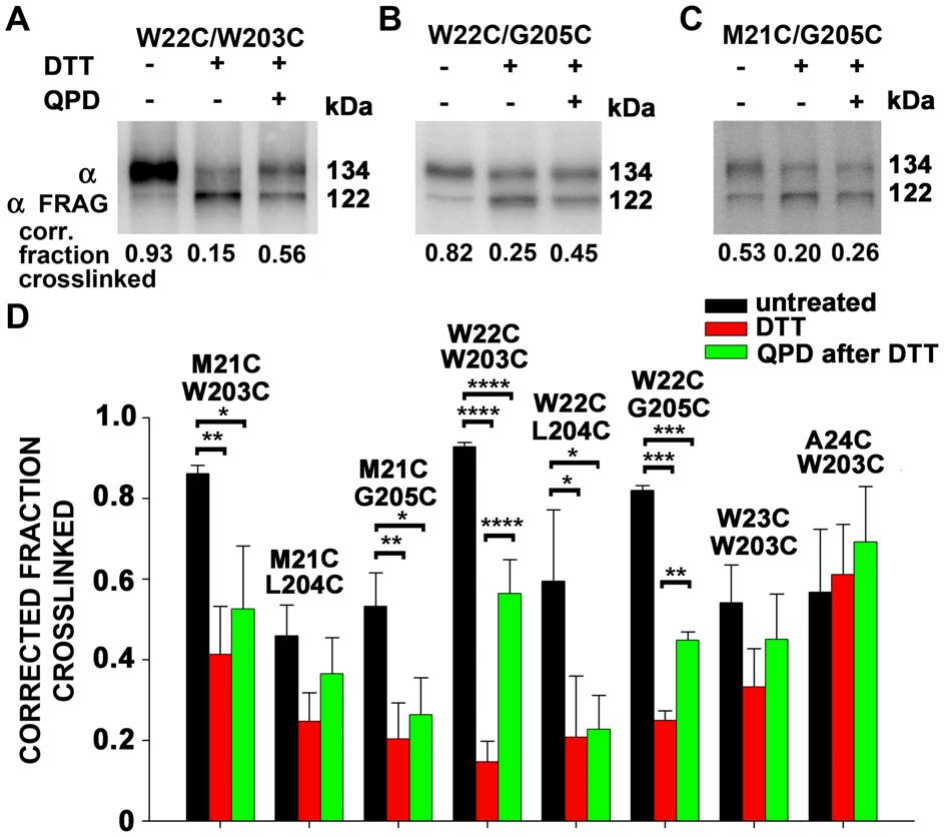
Extents of disulfide bond formation between Cys in S0 and Cys in S4 . (A–C) Cells were transfected with the indicated double-Cys-mutant BK α. After 2 days, the cells were collected, and biotinylated with the impermeant sulfo-NHS-biotin. The cells were divided and were either not further treated, treated with 10 mM DTT, or treated with 10 mM DTT and 40 μM QPD. The conditions were the same as in Fig. 2. Cells were lysed. Solubilized BK α was captured on Neutravidin beads, cleaved with HRV-3c protease between S0 and S1, electrophoresed, and immuno-blotted with an anti-BK α-C-terminal-epitope antibody. The extents of crosslinking were calculated from the relative integrated densities of the full-length α band and the truncated (Frag) α band, corrected by the efficiency of HRV-3c cleavage, determined individually for each Cys pair in each experiment (not shown). The efficiencies of cleavage were approximately 70%. N = 2–4. Mean + SD. N = 2–4 experiments, each with duplicate determinations. * P<0.05, **P<0.01, *** P<0.001, ****, P< 0.0001 by one-way Anova followed by Tukey’s post-hoc analysis.

The biochemically determined fractions of crosslinked and of free Cys are needed to estimate separately the functional effects of a disulfide between two Cys and of the mutation to a Cys per se [23]. Consistent with the functional effects of DTT and QPD on W22C/W203C, the reduction by DTT and reformation of this disulfide by QPD were the most extensive among all the tested pairs (Fig. 4A,D). In the case of W22C/G205C, there was almost as much reduction of the disulfide by DTT and less but still significant restoration of the disulfide by QPD. In this case, however, only DTT affected V_50_, not QPD (Fig. 2C). In M21C/W203C, M21C/G205C, and W22C/L204C, DTT reduced the disulfide but there was either an insignificant or no restoration of the disulfide by QPD (Fig. 4C,D), which accounts for the absence of a QPD-induced shift in the V_50_ (Fig. 2C). For the double mutants, W23C/W203C and A24C/W203C, DTT did not reduce the disulfides (Fig. 4D), which corresponds with the absence of a DTT-induced shift in the V_50_ (Fig. 2C).

QPD can act only on reduced Cys and only if it has access to the Cys. Furthermore, because only the thiolate is reactive, the Cys must be in a sufficiently polar environment to ionize appreciably. Obviously, Cys that were crosslinked endogenously must spend some time as neighbors in the reduced state. That time could be different in the surface membrane and in the endoplasmic reticulum. At least one pair of Cys, W22C and W203C, appear to be aligned with one another in channels both in the ER and at the surface and in both the deactivated and activated conformations.

### Disulfide crosslink between R20C and W203C

**Figure 5:**
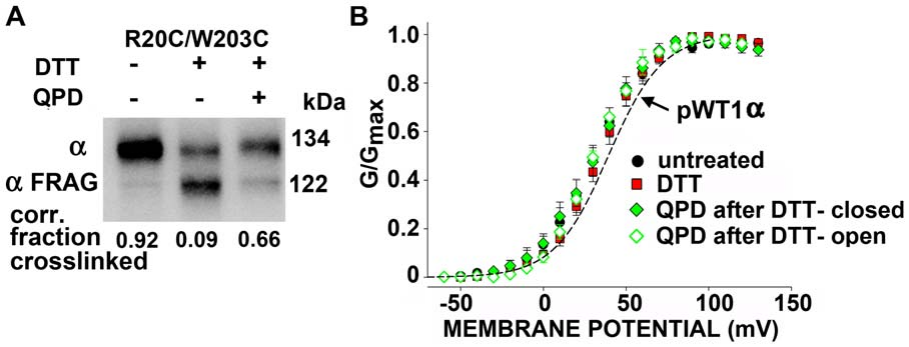
Disulfide bond formation between R20C flanking S0 and W203C in S4. (A) Intact cells transfected with BK αR20C/W203C were treated and analyzed as in Fig. 4. The extents of crosslinking, corrected for the efficiencies of HRV-3C cleavage, are shown below the blots. N = 2. (B) Normalized G-V curves of R20C/W203C either untreated (black), after 10 mM DTT for 5 min (red), after DTT and 40 μM QPD for 2 min, applied in the closed state (filled green diamond), or after DTT and QPD applied in the open state (open green diamond). Fits of a Boltzmann equation were to the means and SD of normalized conductances from separate patches. The dashed line indicates the G-V curve of pWT1 α channels. The pipette solution contained 10 μM Ca^2+^. N = 3–6.

Based on quenching of a fluorophore covalently attached to R20C in the flank of S0, Olcese and co-workers [26] concluded that R20C and W203 are further apart in the activated state than in the deactivated state. We found that R20C and W203C were endogenously crosslinked to greater than 90% (Fig. 5A). Furthermore, this disulfide was almost completely reduced by DTT, and the reduced Cys were extensively reoxidized by QPD. Notwithstanding, neither the initial disulfide crosslink, nor its reduction, nor its reformation shifted the G-V curve (Fig. 5B). Although R20 may move relative to W203 during activation [26], the prevention of such movement by a disulfide bond did not alter the relative stabilities of the activated and deactivated states.

### Orientation of S0 relative to S4 and TM2

**Figure 6:**
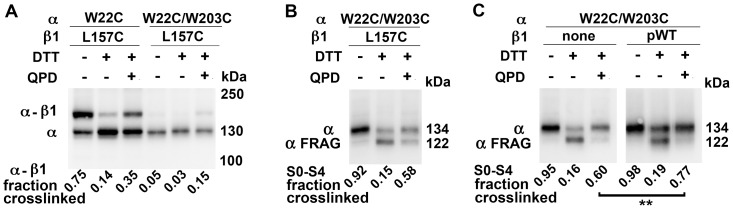
Competition between W203C in S4 and L157C in TM2 for crosslinking to W22C in S0. (A) Cells were transfected with indicated α and β1 subunit mutants. In A, the extent of formation of disulfide-crosslinked α and β1 was determined. In B and C, the extent of formation of an intra-α-subunit disulfide between S0 and S4 was determined. In all cases, three conditions as described in Fig. 4 were analyzed: untreated, reduced with DTT, and reduced with DTT and reoxidized with QPD. Mean + s.e.m. N = 3. **P = 0.01, paired Student’s *t* test.

We previously showed that the extracellular end of S0 lies between S4 and β1 TM2 [25]. Although W22C in S0 readily crosslinks to W203C in S4, W22C can also crosslink to L157C in TM2. In this case, the crosslinked product is a heterodimer of α and β1 with an apparent MW of 160 kDa, which accounts for ∼75% of all α (Fig. 6A, lane 1). This crosslink is reducible by DTT and can be substantially reformed on the cell surface with QPD (Fig. 6A, lanes 2 and 3). In the simultaneous presence of W203C, however, very little α- β1 is crosslinked either endogenously or by QPD after reduction by DTT (Fig. 6A, lanes 4–6). By contrast, W22C and W203C are endogenously crosslinked just as extensively in the presence of L157C (Fig. 6B, lane 1) as in its absence (Fig. 6C, lane 1). Reduction of this crosslink with DTT and its reoxidation by QPD also proceed nearly to the same extent in the presence of L157C (Fig. 6B, lanes 2 and 3) as in its absence (Fig. 6C, lanes 2 and 3). This last result argues against the possibility that the preference of W22C for W203C compared to L157C is due to this crosslinking taking place in the ER before β1 containing L157C associates with α containing W22C and W203C. The QPD result shows that W22C prefers W203C even on the cell surface in a complex with β1 L157C (Fig. 1C).

It is possible that the juxtaposition of W22C and W203C is enhanced by the docking of β1 between S0 of one VSD and S1 and S2 of the adjacent VSD. After reduction of the W22C-W203C disulfide by DTT, its reoxidation by QPD is on average 26% greater in the presence of β1 than in its absence (Fig. 6C; P = 0.01).

**Figure 7:**
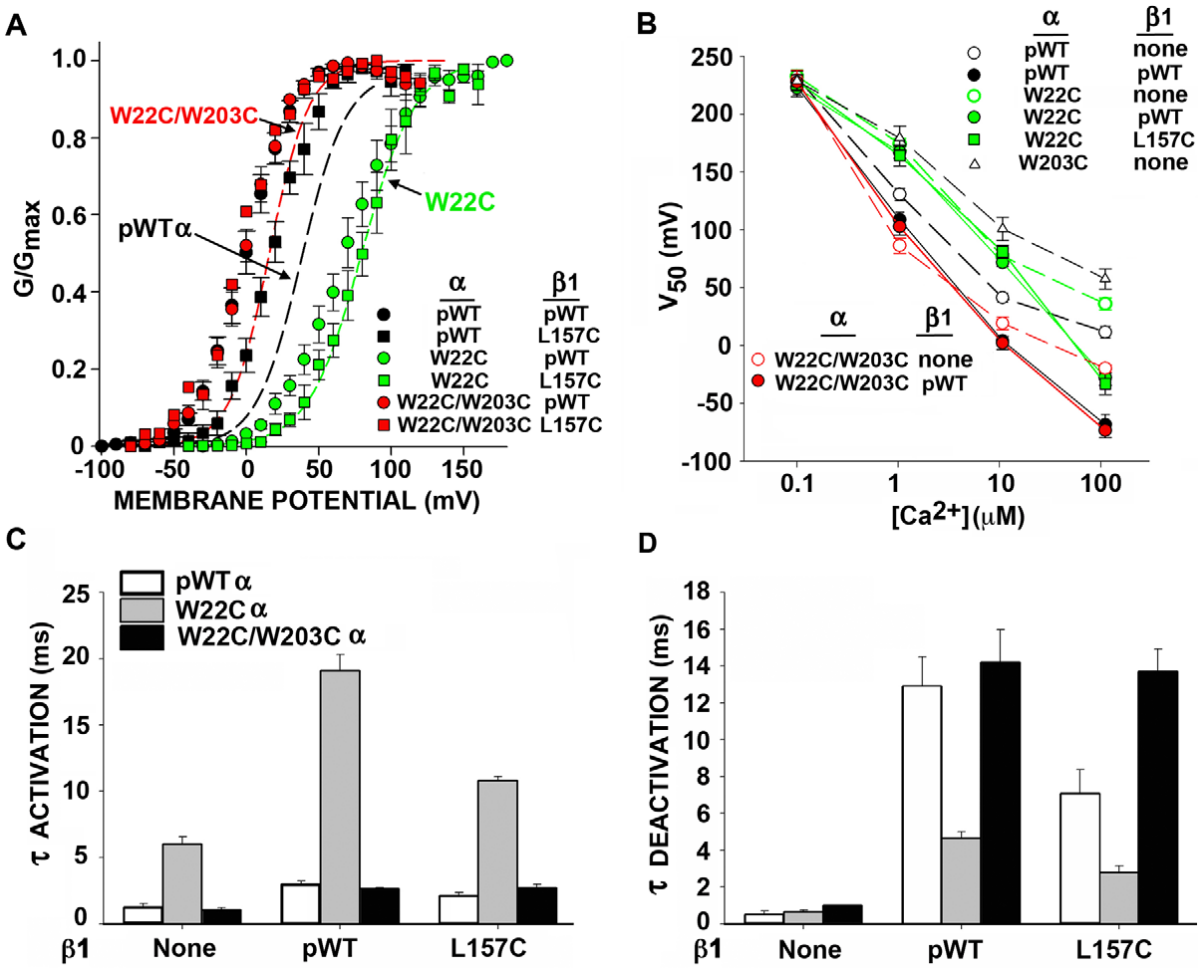
Effects of the mutations W22C in S0, W203C in S4, and L157C in TM2, singly and in combination, on BK channel function. (A) Normalized G-V curves. The data for the combinations of α and β1 are shown as individual points. The data for α alone are shown as fitted curves. (B) V_50_ as a function of [Ca^2+^]_IN_ for various combinations. (C) Tau_ACTIVATION_ and (D) Tau_DEACTIVATION_ for various combinations of pWT1 α or mutant α and β1 or β1 L157C or without β1. Errors are SEM. N = 3–9.

Consistent with β1 acting in part to stabilize a particular interaction of S0 with S4 is the result that the effect on V_50_ of crosslinking W22C and W203C is similar to the effect of incorporating β1 in the channel complex (Fig. 7A,B; Fig. S1). In the first case, the V_50_ of the channel formed by α W22C/W203C alone is shifted negatively about 20 mV (at 10 μM Ca^2+^), whereas β1 shifts the V_50_ of pWT1 α negatively about 30 mV. Adding β1 to α W22C/W203C shifts the V_50_ negatively only by 10 mV, so that the G-V curves of the pWT1 α + β1 complex and the αW22C/W203C + β1 complex superimpose. The same result is obtained with the mutant β1 L157C. As seen above, L157C does not interfere with the formation of the W22C-W203C crosslink. The W22C-W203C crosslink mimics the effect of β1 on the G-V curve but not the slowing by β1 of activation and deactivation (Fig. 7C,D; Fig. S1). β1 has approximately the same effects on the rates of activation and deactivation in complex with pWT1 α and in complex with α W22C/W203C.

That αW22 plays an important role in α-β1 interaction is indicated by the effect of the single mutation W22C (no disulfide). This mutation blocks the negative shift in V_50_ by β1, completely at 10 μM Ca^2+^ and partially at 100 μM Ca^2+^ (Fig. 7B).

## Discussion

### Structural relationships of S0, S4, and TM2 from the extents of crosslinking

Almost all pairs tested among Cys substituted in the first turns of the three α helices, S0, S4, and TM2 show some crosslinking. This implies that even within the membrane the first turns of the helices are somewhat flexible. Nevertheless, there were clear patterns in the extents of crosslinking from which may be inferred the most common relative orientations of the helices (Fig. 1C). Averages of the extents of crosslinking of each substituted Cys in S0 with each of the four Cys substituted in S4 are consistent with M21C and W22C in S0 facing S4 (Table S1). Similarly, averages of the extents of crosslinking of each of the Cys in S4 with each of the four Cys in S0 are consistent with W203C in S4 facing S0. W22C and W203C are crosslinked to a high degree both endogenously, presumably in the ER, and by QPD at the cell surface after their reduction. Although W22C in S0 can form a disulfide with either W203C in S4 or L157C in TM2 of β1, when both W203C and L157C are present, the crosslinking is almost exclusively with W203C (Fig. 6). These findings imply that W22 in S0 and W203 in S4 are aligned with one another in both the absence and presence of β1.

Fluctuations in the relative orientations of these helices or even in their secondary structures were more evident from the endogenous crosslinking than from that induced by QPD at the cell surface. To the extent that the endogenous crosslinking is due to protein disulfide isomerases (PDIs) in the endoplasmic reticulum, these also function as chaperones and could promote some abstraction of the helices from the membrane and their partial unfolding [22]. QPD on the other hand is a relatively bulky, doubly positively charged reagent, which is unlikely to spend much time in a hydrophobic and/or crowded environment. Despite the deviations the preferred structures required by some of the crosslinks, the channels bearing these crosslinks were transported to the cell surface and were functional. These experiments were performed in a pWT background, in which Cys14 and Cys141 were mutated to Ala, resulting in the loss of the endogenous disulfide bond. The mutation of one or both of Cys14 and Cys141 to Ala has no detectable effect on the function of the α subunit. We previously showed that the activation and deactivation kinetics and the G-V curve in WT α and in C14A, C141A, and C14A/C141A (pWT α) were all identical [22]. C14A/C141A α (pWT α) and WT α are modulated identically by β1 [23]. Our approach, which relies on endogenous disulfide bond formation within the endoplasmic reticulum of extracytoplasmic (destined to be extracellular) pairs of engineered Cys, would not be possible if native extracytoplasmic Cys were present. These extracytoplasmic Cys could compete for disulfide bond formation with one or both of the engineered Cys, and there is no way of determining the extent of this competition. For unambiguous interpretation of the crosslinking, Cys14 and Cys141 must be mutated.

### Voltage-dependent movements of the extracellular ends of S0 and S4

Several groups using substituted-Cys-accessibility methods [29], crosslinking, or fluorometry on other voltage-dependent K^+^ channels and Na^+^ channels have inferred movements of S4 relative to the bilayer or relative to other TM helices [30]–[39]. In BK channels, as well, voltage-dependent movements of S4 [40]-[42] and, in particular, separation of S0 and S4 [26] have been inferred from perturbations of fluorescent reporters. In addition, Horrigan and co-workers observed voltage-dependent Cu^2+^ binding to residues in S2, S3 and S4, consistent with some rearrangement of the BK voltage-sensor TM helices [43]. We found, however, that nearly complete disulfide crosslinking of R20C in S0 to W203C in S4, two positions that Pantazis et al. [26] inferred separated on activation, had no effect on the V_50_ for opening. This implies that separation of these two residues and, hence, of the extracellular ends of S0 and S4 might occur but is not required for activation. Furthermore, our finding that there was no difference in the rate constants in the activated and deactivated states for the induced reformation of the disulfide between W22C and W203C at the cell surface argues against much relative movement (Fig. 3). We did not evaluate the degree or significance of the interaction between the native W22 and W203 and their change in relative positions, or lack of it, during activation.

Despite the similar relative dispositions of W22C and W203C in the activated and deactivated states, paradoxically the crosslink shifted the V_50_ for opening negatively and hence stabilized the open state at Ca^2+^ of 1 μM or greater. It is possible that this interface is normally more constrained in the activated state than in the inactivated state and that by pre-constraining the interface, the disulfide removes an entropic cost of activation and thereby stabilizes the open state. It is not readily apparent why the V50’s for W22C and W22C/W203C are not shifted to more positive and negative potentials respectively at nominally 0 Ca^2+^. The size of the shift may increase with increasing Ca^2+^ even with a change only in the voltage sensor equilibrium constant in the context of the dual allosteric model [44].

Some but not all crosslinks across the S0-S4 interface favor the open state. For example, the crosslinks of both R20C and M21C in S0 to W203C in S4 favored the closed state. Also, in the flanking regions, some crosslinks between S0 and the four-residue S3–S4 loop stabilized the open state, some stabilized the closed state, and only a few had no effect at all [22]. Crosslinks can of course distort the structures of the crosslinked segments as well as constrain their relative movement. Nevertheless, that even a few crosslinks have little effect on V_50_ indicate that either the top of S4 does not move much or that S0 and S4 normally move together.

Compared to other voltage-gated K^+^ channels, the BK channel has a smaller gating charge, just 0.9 *e* per subunit [45]. Moreover, the residues contributing to the gating charge are decentralized, consisting of two residues in S2 (D153 and R167), one in S3 (D186), and only one in S4 (R213), this last residue contributing one-half of the gating charge [46]. Nevertheless, during activation R213 must move relative to the electric field. A possibility is that S4 and S0 move together across the membrane parallel to the electric field. Another possibility is that S4, or at least its lower half, changes its angle to the electric field with the extracellular end of S4 constrained by its interaction with a stationary S0.

### Inferences about the function of the S0-TM2 interface

Bao and Cox [47] found that β1 stabilizes the activated state, shifting the V_50_ for gating current negatively. We now find that the crosslink between W22C in S0 and W203C in S4 shifts the G-V curve of BK α 20 mV in the negative direction, similar to the 30-mV shift due to β1 acting on pWT1 α. Addition of β1 to α with the W22C-W203C crosslink shifts the G-V curve only another 10 mV to the left, so that it superimposes on the G-V curve of pWT1 α plus β1. Although similar shifts do not necessarily imply similar mechanisms, these results are at least consistent with the hypothesis that β1 TM2, binding to S0, constrains the interface between the extracellular ends of S0 and S4 and that this constraint stabilizes the activated state. Also, W22 may make a significant contribution to the postulated constraint of S0 and S4 in the activated state, because mutation to the smaller Cys residue increases V_50_ and suppresses the negative shift in V_50_ due to β1 (Fig. 7A,B).

The results here directly probe only the first helical turns of the S0-S4 and S0-TM2 interfaces. The extent of the contact surface between S0 and S4 below the first helical turns is not known. It might include, however, F25, L26, and S29, the mutations of which to Trp shifted V_50_ by +40 to +80 mV [44]. In a regular helix, these residues would be on the same side of S0 as W22. All other tested mutations of the residues of S0, including W22A, had much smaller effects. These results are consistent with the importance of the S0-S4 interface in determining the relative stabilities of the activated and deactivated states of the VSD. Also, the extent of the contact surface between S0 and TM2 below the first helical turns is not known. The paths of the three helices, TM2, S0, and S4 through the membrane would be revealed by the propensities of substituted Cys at their intracellular ends to form disulfide bonds with each other and with the other TM helices in α and in β1. Other regions of β1, such as the N-terminal and C-terminal tails [48]–[50] and the extracellular loop [51], [52], also contribute to the modulation of the channel. The functional roles of the interfaces between TM1 and each of S1 and S2 [25] remain unexplored.

## Supporting Information

Figure S1 **Macroscopic currents conducted by pWT1 α, W22C α and W22/W203C α alone or co-expressed with either pWT β1 or β1 L157C.** Currents were activated by depolarizing steps from a holding potential of −100 mV and deactivated by repolarization to −100 mV. [Ca^2+^]_IN_ was 10 μM.
(TIF)

Table S1 **Extents of disulfide crosslinking of S0 to S4.** The residues substituted by Cys are shown.
(PDF)
