# Supplementary figures and images for: Orientations and Proximities of the Extracellular Ends of Transmembrane Helices S0 and S4 in Open and Closed BK Potassium Channels
Source: PLoS One. 2013 Mar 5;8(3):e58335. doi: 10.1371/journal.pone.0058335 (PMC3589268; doi:10.1371/journal.pone.0058335)

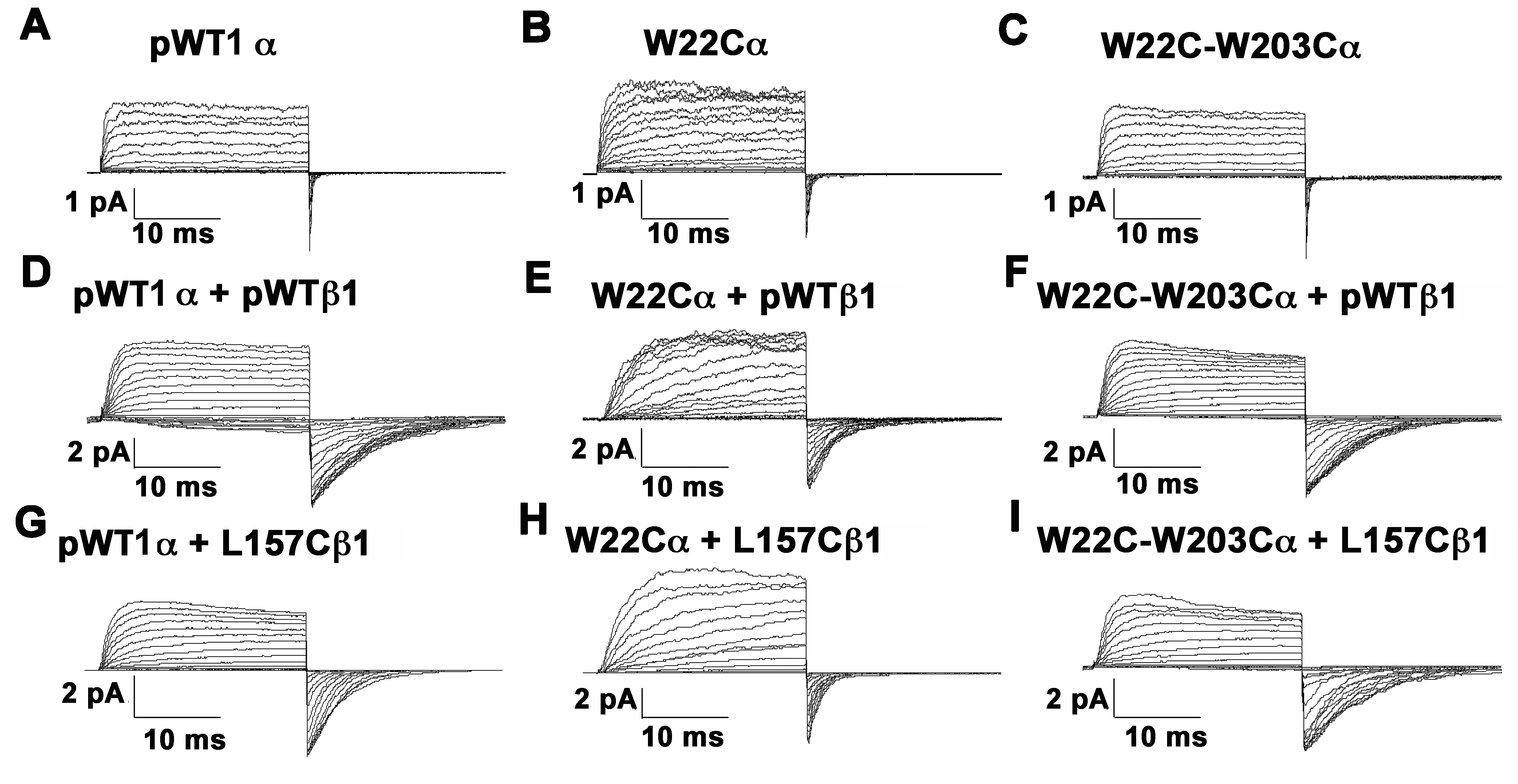

Supplement: Figure S1 — Macroscopic currents conducted by pWT1 α, W22C α and W22/W203C α alone or co-expressed with either pWT β1 or β1 L157C. Currents were activated by depolarizing steps from a holding potential of −100 mV and deactivated by repolarization to −100 mV. [Ca2+]IN was 10 μM. (TIF) [file pone.0058335.s001.tif]
