# Supplementary material for: Orientations and Proximities of the Extracellular Ends of Transmembrane Helices S0 and S4 in Open and Closed BK Potassium Channels
Source: PLoS One. 2013 Mar 5;8(3):e58335. doi: 10.1371/journal.pone.0058335 (PMC3589268; doi:10.1371/journal.pone.0058335)
Supplement: Table S1 — Extents of disulfide crosslinking of S0 to S4. The residues substituted by Cys are shown. (PDF) [file pone.0058335.s002.pdf]

	**S0**	M21	W22	W23	A24	**MEAN**	SD
**S4**							
W203		0.62	0.72	0.39	0.49	**0.56**	0.14
L204		0.37	0.51	0.12	0.12	**0.28**	0.19
G205		0.43	0.63	-0.04	0.04	**0.27**	0.32
I206		0.04	0.12	-0.05	0.05	**0.04**	0.07
**MEAN**		**0.37**	**0.50**	**0.11**	**0.18**		
SD		0.24	0.26	0.21	0.21		

Table S1. **Extents of disulfide crosslinking of S0 to S4.** The residues substituted by Cys are shown.
